# Concealment and discovery: The role of information security in biomedical data re-use

**DOI:** 10.1177/0306312718804875

**Published:** 2018-10-15

**Authors:** Niccolò Tempini, Sabina Leonelli

**Affiliations:** Egenis, The Centre for the Study of Life Sciences, Department of Sociology, Philosophy and Anthropology, University of Exeter, Exeter, UK; Egenis, The Centre for the Study of Life Sciences, Department of Sociology, Philosophy and Anthropology, University of Exeter, Exeter, UK; School of Humanities, The University of Adelaide, Adelaide, SA, Australia

**Keywords:** big data, biomedicine, databases, data infrastructures, data re-use, information security

## Abstract

This paper analyses the role of information security (IS) in shaping the dissemination and re-use of biomedical data, as well as the embedding of such data in material, social and regulatory landscapes of research. We consider data management practices adopted by two UK-based data linkage infrastructures: the Secure Anonymised Information Linkage, a Welsh databank that facilitates appropriate re-use of health data derived from research and routine medical practice in the region, and the Medical and Environmental Data Mash-up Infrastructure, a project bringing together researchers to link and analyse complex meteorological, environmental and epidemiological data. Through an in-depth analysis of how data are sourced, processed and analysed in these two cases, we show that IS takes two distinct forms: epistemic IS, focused on protecting the reliability and reusability of data as they move across platforms and research contexts, and infrastructural IS, concerned with protecting data from external attacks, mishandling and use disruption. These two dimensions are intertwined and mutually constitutive, and yet are often perceived by researchers as being in tension with each other. We discuss how such tensions emerge when the two dimensions of IS are operationalized in ways that put them at cross purpose with each other, thus exemplifying the vulnerability of data management strategies to broader governance and technological regimes. We also show that whenever biomedical researchers manage to overcome the conflict, the interplay between epistemic and infrastructural IS prompts critical questions concerning data sources, formats, metadata and potential uses, resulting in an improved understanding of the wider context of research and the development of relevant resources. This informs and significantly improves the reusability of biomedical data, while encouraging exploratory analyses of secondary data sources.

## Information security, data-driven discovery and biomedical data infrastructures

Contemporary discourse on information technology and data infrastructures typically interprets the concept of ‘information security’ (IS) as referring to the set of practices aimed at controlling and supporting the access and use of information within a given organization. These practices encompass the development and application of procedural and technical tools, including policies (e.g. information governance), organizational processes (e.g. information governance review, anonymization procedures), technologies (e.g. security and enforcement tools), and other organizational arrangements. Understanding the relationship between IS and scientific process is paramount to understand how infrastructures devoted to facilitating data re-use can fulfill their goals. Security requirements and information governance in particular exert a strong influence on the trajectories and outcomes of data sharing efforts, in ways that researchers often perceive to be at odds with the emphasis on exploratory research typically invoked by advocates of ‘open’ and ‘big data’ science (e.g. Hey et al., 2009; Mishra et al., 2015; National Research Council, 2009; Sansone et al., 2012). Authoritative reports point to the tension between attempting to free data access from any restrictions and making sure that the shared data are of high quality, conform to safety and security standards (Boulton et al., 2012; Science International, 2015). Policymakers have developed policies and platforms to foster open science while mitigating the risks associated with making data accessible on a large scale (Department of Health, Data Sharing and Cyber Security Team, 2017; Leonelli, 2016b). These contributions have highlighted how security and ethics are deeply linked with the successes and failures of data re-use and data-intensive methods, yet the nature of the relationship has not been clarified.

Biomedical data are perhaps the paramount case for this kind of tension. General ethical principles to safeguard patients and protect their wellbeing, privacy and confidential information are widely agreed by practitioners to be conditions of possibility for their interventions. Security measures and information governance procedures can be put in place to prevent such misguided uses of data, but they are often perceived by researchers as constraining users’ freedom to analyze the data as they see fit (Richards et al., 2015). Biomedical data are typically difficult to access and use, due, among other factors, to legal uncertainty, the complexity of ethics reviews, potential commercial interests, and uncertainty around the processes, standards and practices through which data have been generated (Hogle, 2016). If research is multi-sited, concerns emerge for ethics review processes to maintain consistency in judgement while avoiding duplication of efforts (Dove et al., 2016). These issues are compounded by the shift towards the digital, which has dramatically increased the ease with which data can be copied, circulated, corrupted and leaked.^1^

In this paper, we investigate this perceived tension between the epistemic goals and priorities of biomedical research, and the constraints associated with the logistics and ethical concerns around health data storage, processing and anonymization. Considering cases from the biomedical domain thus has the advantage of building on a large body of scholarship concerned with data privacy and security issues, which are recognized as critical to the management of personal and confidential data extracted from the study and treatment of human subjects (Mittelstadt and Floridi, 2016). Scholars have mapped the ways in which privacy concerns are shifting form (Aicardi et al., 2016; Floridi, 2014; Taylor et al., 2017) as a result of the technological, methodological and organizational innovations of big data science (Dove et al., 2015); some argue that privacy frameworks should be updated to include protections for groups and local communities (Floridi, 2014; Prainsack and Buyx, 2017). In science and technology studies, issues of security are presented as important for a reasoned and risk-sensitive take on the organization of a data-intensive research infrastructure (e.g. Demir and Murtagh, 2013; Dove et al., 2016; Ossorio, 2011). Dove et al. (2015), discussing ethical and legal issues raised by cloud computing in genomics research, offer four ‘points to consider’ for structuring the negotiation and organization of infrastructural arrangements: data control, data security, confidentiality and transfer, and accountability. Burton et al. (2015) stress how the perceived tension between protection of privacy and the conduct of research is a result of the dichotomy between the moral principles of individual rights protection and the public good (also Floridi, 2014). They emphasize how this dichotomy is continuously tested in a changing societal context: infrastructure managers need to be concerned not only with how to build trust, but also with how to maintain it. Burton and colleagues advocate a responsive and dynamic approach to trustworthiness as the only strategy for keeping an infrastructure abreast with changing norms and values. Importantly for the argument presented in this paper, they maintain that a concern with the protection of privacy needs to be matched by an equal concern with the integrity of the data, which can be compromised by some particularly protective approaches.

With its strong emphasis on the challenges posed by confidentiality, this scholarship provides a strong platform to reflect on the relationship between IS and scientific process. At the same time, such work tends to conceptualize security as an indispensable yet external condition to using data for research, and pays little attention to the ways in which IS regimes are an integral part of regimes of knowledge production that include guiding principles, goals, methods and tools. The tendency to ‘externalize’ IS is deeply rooted in the history of security practices, which have largely been developed in relation to the needs and priorities of enterprise systems development, and the cost-benefit risk evaluations of business enterprise users.^2^ One of the most typical applications of IS consists in addressing the risk of cyberattacks and leaks, with practitioners focusing largely on the new forms of attack of the age of internet networking, where a remote attacker can reach an organization’s digital doorstep in a few steps, and the complex risks involved in data breaches; as a result, computer security and national security have become increasingly intertwined (e.g. Baskerville et al., 2014; Nissenbaum, 2005). In those cases, security has typically been conceptualized as relative to an external environment (Tempini, 2016).^3^ However, as we demonstrate here, IS can and often does undergo considerable modification to fit the specific contexts and goals of scientific work, resulting in practices that are not merely applied to knowledge production but are deeply intertwined and co-produced with it. To this aim we focus on the role played by IS in the stewardship, dissemination and re-use of biomedical data for research purposes. Based on the detailed empirical study of two specific cases, we show that IS is not external to scientific research, but rather is a constitutive part of the process of knowledge-making in biomedicine. This happens because of the interplay between two forms of IS: *epistemic* and *infrastructural*. Demonstrating the purchase of this conceptualization is the core concern of this paper.

*Epistemic information security*, or EIS, concerns the extent to which data can be guaranteed to be reliable and trustworthy as evidence for knowledge claims, and thus as credible and reusable sources of information about the world. This requirement is described by Wylie (2017) as involving the expectation that ‘each line of evidence – including its anchoring facts or observations and the warrants for their interpretation as evidence – must be credible in its own right’. EIS involves making data defensible against potential accusations of data fabrication, tampering or mishandling, while at the same time managing them to preserve their re-usability in different situations and for a long time after their generation. The main risk managed by EIS is that of undermining the evidential value of data, for instance by failing to provide appropriate metadata to support data analysis, structuring data collections in ways that make them hard to search, or editing them for use in specific situations in ways that are not reversible. By contrast, *infrastructural information security*, or IIS, is concerned less with the value of data as evidence, and more with their control and protection as valuable assets. IIS thus involves the defense of a given dataset, and the control over who can access the data, and how. In line with mainstream, outward-looking approaches to data security, the main risk managed by IIS is that of data hacking, unauthorized access and theft.

These two dimensions are highly interdependent in practice and yet they have become associated with different skills, concerns and accountabilities. As we discuss in more detail below, EIS tends to be portrayed as the purview of experimental and clinical researchers. Their main interest is in knowledge production, both in the sense of generating knowledge claims and in the sense of devising novel forms of intervention; their skills and responsibilities are framed accordingly as encompassing the identification and testing of inferences and causal claims, and the development and validation of research methods. IIS is instead associated with the professional roles of software and information engineers who are tasked with enforcing boundaries between different human activities. Their work is construed as guaranteeing the safety of data from outside interference while at the same time serving the information needs of software users.

This division of labour between EIS and IIS is becoming ever more pronounced and formalized as the tasks involved in pursuing them become more complex and specialized. As a result, EIS and IIS are often perceived to be in tension with each other by the researchers involved. In contrast to this, we challenge the often-assumed polarisation between EIS and IIS, in favour of a more complex and open-ended characterization of their relationship.

Our analysis emphasizes the mutually constitutive relationship between knowledge production and IS in the age of data-intensive, ‘open’, large-scale research, thus challenging the idea of IS as a mere remedy to ‘disturbances’ – such as politics, ethics and hacking attempts – perceived as extraneous to science. In so doing, we take inspiration both from the long-standing tradition in information security literature to associate IS with the preservation of data confidentiality, integrity and accessibility, which is compatible with concerns around data re-use, and from seminal STS work on the boundaries between science, policy and social engagement and the many forms of co-production informing the practices and outcomes of research.

In what follows, we document cases in which security regimes are used to contribute and shape the processes and outcomes of biomedical knowledge production, for instance by prompting researchers to critically question their expectations with regard to data sources, formats, necessary metadata and potential uses, to forge new collaborations, and to account for the wider social context of the research in ways that may enhance the re-usability of data and help prevent future problems.^4^ We focus on the ways in which the complex socio-technical systems of data management operate in response to IS issues; the ways in which data are circulated and re-used in research projects conducted through the infrastructures; and the difference that taking account of IS regimes makes to set-up and outcomes of research projects. We argue that EIS and IIS are not necessarily in conflict with each other: The tensions between these two forms of IS derive from the ways in which researchers and the institutional settings within which they work tackle the division of scientific labour, often rendering data management strategies vulnerable to technical innovations, public controversies or regulatory shifts. When EIS and IIS are combined at all stages of the research process, the loss of freedom related to specific implementations of IS is balanced by the broader applicability and social responsiveness of the data management strategies being devised.

Our empirical exploration is grounded in the study of how biomedical data travel from their site of production to sites in which they are disseminated and re-used, which we call ‘data journeys’ (Leonelli, 2016a). We are particularly interested in journeys that involve a wide variety of data handling practices, and within which data are reformatted, manipulated and adapted to different technologies, habits, institutional settings and goals.^5^ We study ethnographically two data linkage infrastructures for biomedical research: the Secure Anonymised Information Linkage (SAIL), a Welsh databank that aims to facilitate appropriate re-use of health data derived from research and routine medical practice in the region; and the Medical and Environmental Data Mash-up Infrastructure (MEDMI), a project bringing together researchers from the University of Exeter, the London School of Hygiene and Tropical Medicine, the Met Office and Public Health England to link and analyse complex meteorological, environmental and epidemiological data. SAIL is dedicated to fostering the re-use of routine health and administrative data and other previously generated research datasets; while MEDMI is centered on the collection and re-analysis of large environmental and health datasets to foster the integration of evidence available on human health and wellbeing, and their relationship with the environment.

Both projects are leading examples of the developing field of health data linkage methods, which promises to produce more comprehensive health profiles for real world patients than those hitherto accomplished by traditional clinical services or epidemiology – for instance by connecting electronic health records with environmental and weather data, educational and other socio-economic data, and genetic profiles. Linkage opens spaces of research that are unattainable when relying on fewer data sources and traditional comparative methods, but are also widely understood as increasing risk to patient protection and privacy. We chose to focus on SAIL and MEDMI insofar as they both exemplify how the new opportunities offered by data linkage need to be conceptualized and developed hand-in-hand with new methods to control risk, and yet they approach this issue in different ways. The two cases also represent different stages in the development of a large biomedical data infrastructure. SAIL is in many respects a mature infrastructure, which over the past decade grew from a small pilot project into a sophisticated system of data management with a high number of data-rich users and stakeholders. SAIL therefore demonstrates the organizational and technological solutions needed to cover health data for a large population and territory with a long-term perspective. MEDMI, by contrast, is an infrastructure in early stages of development, whose funding and assessment are still tied to a specific project and its expected outcomes. As such, it provides insight on smaller-scale interdependencies and on the challenges involved in developing a radically interdisciplinary meeting point between climate science, social epidemiology, population genetics and environmental research.

We conducted participant observation on the handling and re-use of data through MEDMI and SAIL from March 2015 to January 2017, including 40 semi-structured in-depth interviews with key members and users at all levels,^6^ joint meetings and discussion sessions, and consultation of the extensive archives and publications released by the two infrastructures, with an interest in documenting different perspectives on their functioning, ambitions and outputs. Both SAIL and MEDMI have accumulated considerable institutional memory of the opportunities and challenges involved in processing and analyzing health data, as well as the diverse set of needs and situations for which researchers may need access to those data. These resources are prime sites for fielding questions on the role of IS in relation to epistemic worries such as the trustworthiness, reliability and long-term sustainability of the data in question for use as evidence for knowledge claims. While not initially the focus of our inquiry, information security emerged early on in our investigation as a critical dimension of concern for publicly funded information linkage infrastructures focused on the storage, linkage, (re)analysis and dissemination of human health data.

IS, in both its epistemic and infrastructural forms, is a constructive and constitutive element of epistemic strategies to achieve reliable inferences and dependable datasets in the context of health research through big data. While attention to IS does impose additional constraints on data dissemination methods, thus curtailing unmediated access to data produced by scientific projects and limiting their outputs in the short term, this should not be perceived as a costly inconvenient. Investment in IS can help researchers to develop sustainable and well-curated data pipelines in the longer term, thus improving the accessibility of increasing numbers of sources and tools, facilitating the execution of data analysis and mitigating some of the risks involved in linking vast amounts of health data. Early and continued attention to both EIS and IIS can make data re-use in the long term easier, better grounded and more resilient to sweeping changes in the broader institutional landscape. Scientific research today cannot escape the multiple demands for trustworthiness being advanced all at once.

## The Secure Anonymised Information Linkage (SAIL)

SAIL’s aim is to make possible the re-use, for health research purposes, of routine data generated through public services and of otherwise unavailable datasets generated in the context of individual scientific projects. SAIL was developed within the Health Informatics Research Unit (HIRU) at the University of Swansea in Wales, to build a world-class health research facility for research communities both locally and globally, aiming at repurposing biomedical datasets through their integration in a dedicated digital infrastructure.

SAIL currently hosts billions of data points about approximately four million residents of Wales (Jones et al., 2014) and supports researchers distributed all over the globe, yet its large-scale operations had a humble beginning. In 2006 a grant funded by the Wales Office for Research and Development (now Health and Care Research Wales – see below) supported the creation of HIRU, with the aim of developing systematic approaches to foster the re-use of data. System development activity quickly ensued, to eventually roll out a pilot after nine months. The pilot system aggregated data from the neighbouring area of Swansea, a catchment of almost 300,000 people, and included the data from all local general practitioners, the hospital, government social services departments and NHS Wales Informatics Services (NWIS), the latter holding national-scale data. By 2007, the system acquired its current name and was re-oriented towards becoming a national (Welsh) scale research infrastructure (Ford et al., 2009; Lyons et al., 2009). The management worked to bring in more datasets from GP practices, hospitals and registries all over Wales; to date, the staff estimates it holds 70–80% of GP practice data across the region. The scale of SAIL is significant, with almost £4 million core funding from Health and Care Research Wales^7^ allocated to the resource since its formal establishment in 2007, and over fifteen members of staff directly employed for the infrastructure, as well as many collaborators employed by Swansea University Medical School.

To further increase the scale of the system, and multiply the number and kinds of data providers, was a complex task. Given the amount of sensitive information about every resident of Wales that such a scientific enterprise aims to hold, the SAIL infrastructure needed to robustly address concerns on privacy and the potential harm that misuse or misinterpretation of the data can generate. A considerable and, for research purposes, very important proportion of the data handled by SAIL is collected from individual GP practices, where practitioners are invested with the duty of protecting patient records. SAIL management thus had to address a diverse range of sensitive confidentiality concerns held by each and every data provider. This made management realize that in order to enable data sharing and research, SAIL needed first and foremost to develop technological and organizational processes to *source* and *manage* data in ways that are widely recognized to be *reliable* and *trustworthy*. Indeed, when discussing their role in acquiring externally produced data and overseeing their use by researchers, SAIL infrastructure managers describe their duties as data ‘custodian’ and ‘guardian’, with responsibilities to guarantee the security of the research environment and maintaining the trust that allows them to keep sourcing data, attract researchers, and dispel risk of misuse.

These were the guiding principles on which basis SAIL managers envisaged a set of complex solutions that facilitated centralized access to the datasets in their possession for the purposes of research use. Concentrating such (unprecedented) amounts of information about an individual is a high-risk proposition, and laws and regulations abound to prevent the use of data to harm individuals. SAIL infrastructure has developed over the years to dissipate and control for the risk of data breaches and misuse, scaffolding a number of technical and procedural solutions over several tiers in order to reduce the risk related to the activities of SAIL staff, collaborating researchers and other external actors.

Since its early stages, the infrastructure needed to withstand scrutiny of both its ability to protect the data from unauthorized access and illicit uses (IIS), and of the reliability and accountability of its processes of data linkage to provide evidence in scientific investigations (EIS). Already in the pilot, a *split-file approach* to data management – SAIL’s current secure linkage method and hallmark – was developed to be able to transfer clinical information at individual record resolution while increasing its security against patient protection risks (see Ford et al., 2009; Jones et al., 2014). This method allows for the selection and encryption of the most important personal identifiers, such as demographic data, before they reach the SAIL databank (so that SAIL never actually hold these highly sensitive data). The resulting record is still usable to track and link individuals across different datasets, in order to reconstruct a comprehensive clinical history of Welsh residents as they move over time across different institutions, organizations and places, accessing different services and interacting with health professionals. The method is designed to support trust by stakeholders, by increasing protection against unauthorized attacks and uses of the data, while simultaneously employing a data management strategy that is accountable and tries to least interfere with the range of uses to which the data can be put.

For this linkage method to work, it requires the collaboration of an independent organization, which takes the role of a ‘trusted third party’, to handle and transform part of the sensitive information so as to make it impossible for anyone (trusted third parties or infrastructure managers) to hold the entirety of the record. In practice, this means that, when making a submission to SAIL, data providers have to separate demographic information (fields for NHS number, name, date of birth, gender, address and postcode) from clinical information data fields (for instance, observed clinical signs, reported symptoms, prescribed treatments, lab results, blood pressure and other measurements, and so forth). The demographic data are sent to the NHS Wales Informatics Service (NWIS), which acts as a trusted third party. Clinical data are sent to SAIL. A SAIL-developed algorithm and software application supports the execution of the task.

Upon reception of the demographic data, NWIS executes a (validated) matching algorithm to perform the linkage. The algorithm aims to identify ‘who’s who’ by comparing the demographic information and tracing the same individual across different datasets. If an individual’s demographic data have never been submitted before, they cannot be matched and are simply anonymized into alphanumerical strings;^8^ the anonymized alphanumerical strings are then sent to SAIL, while the trusted third party keeps the record of what original demographic data correspond to what transformed alphanumerical strings. If, instead, new demographic data are matched with previously processed demographics, the existing de-identified alphanumerical strings are sent to SAIL.

SAIL receives the data in a dedicated loading area restricted to technical support staff, who run a series of batch processes to ensure the integrity and auditability of the data, including version numbering. They flag eventual data quality issues that may require discussions with the trusted third party or the data providers. The process of anonymization itself can make the inspection of a new batch of data more complicated. The correction of errors or discrepancies can become very lengthy and complex to coordinate.^9^ Once these issues are resolved, standardized data are further masked and re-encrypted to be integrated in the SAIL base pool of data, which are then available to SAIL analysts for re-use and collaboration in research projects. Through this process, SAIL can then match and link alphanumerical strings across datasets, and know when anonymized clinical records belong to the same person, without holding plain-text demographic details of any individual. Through multiple checks and processes, the data are protected from eventual wrongdoing by trusted third party, SAIL or research project actors, as well as ‘external’ attackers.^10^

An individual record linked over several datasets through such system offers an informative clinical data landscape, while also implementing filters to protect the anonymity of the individual. The range of potential uses of individual datasets are dramatically extended as they are combined with other datasets, to open a new space for scientific investigations. This is possible through sophisticated security measures that at once guarantee the accountability of the re-use processes with respect to protection against attacks, and the reliability of the data sharing processes in keeping the evidential value of the data from being compromised. Within such a system, the risk of re-identification is a moving target, and requirements need to change and grow more complex over time as the infrastructure is developed to host more data, and attract increasingly diverse and numerous data users. Accordingly, the IS regime needs to be continually reviewed and updated; in SAIL this has resulted in a number of additional technical and procedural approaches, which we group in three categories in what follows.

### Formal roles and institutionalized expertise

First, SAIL staff has been split between infrastructure- and research-facing. Infrastructure staff, a category that includes IT and data warehouse managers and developers, are entrusted with the highest level of access rights, necessary to perform deep ‘backbone’ operations on the data infrastructure. They manage the entirety of the data stored in the SAIL databank (after first-pass anonymization), execute additional levels of anonymization that further secure data against re-identification, check the integrity of the datasets that are sourced and stored, and surveil the system for illicit activity. They are not involved in the use of data for specific projects, so as to be as free as possible from conflicts of interest and to increase the trustworthiness of the system.

Research-facing staff, such as data analysts, are considered data users because they participate in research projects. Analysts specialize in a selection of the available datasets, which they come to know in intimate detail, and learn about new datasets: New datasets are always incoming, so not all relevant information for planning a research project is available on the website. Analysts collaborate with researchers to design and execute each research project (more on this below). They are a first point of contact for a researcher interested in working with SAIL data.

Analysts access data that have already been anonymized twice, making it much more difficult to combine it with data held at the trusted third party to re-identify individuals. They construct an extract of the dataset, to be made accessible to the collaborating researchers, which contains only the data that the individual research project is understood to need for its research purposes; and they translate the extract into a format with which statisticians are familiar (e.g. SPSS, R). This extract is processed by the analyst to anonymize demographic variables a third time, to further reduce the risk that a collaborating researcher (or someone in their stead) who uses SAIL data multiple times for different projects may end up combining the data and re-identifying individuals.

Most importantly, in their role as ‘mediators’ between SAIL users and SAIL data collections, analysts have the opportunity to develop knowledge about what kinds of data might be missing, how certain medical events might have been coded differently between two sets to be compared, and many other factors that shaped the way in which the data were generated. Analysts curate the documentation of the datasets in which they specialize (since datasets as they are shared by donor organizations are often poorly documented). As we see in the next sub-section, their in-depth knowledge is fundamental to the choice of methods of data analysis and controls for biases that are well-suited to the specific datasets at hand – a particularly delicate requirement, given that routine datasets such as that collected in SAIL are often ridden with pitfalls. In some cases, analysts support a research project throughout, from the definition of a research question, to the execution of the linkage and contribution to the analysis and the writing up of methods and results.

### Procedural solutions

Second, a series of procedural solutions have been put in place to govern the use of data. The most important is the Information Governance Review Panel (IGRP), which helps to review, guide and monitor researchers’ uses of the data, and includes external stakeholders such as patient representatives and medical association representatives. This process makes it impossible for any individual researcher to access all of the data stored in SAIL at once. Instead, researchers wishing to access SAIL data are asked to spell out their research needs and goals, which are subsequently vetted by the IGRP.

The first step for a researcher aiming to work with SAIL data is to undergo a scoping review, which is conducted in collaboration with management and senior analysts to analyse the proposal’s feasibility. SAIL staff help researchers to specify the research project in detail, including evaluation of the costs, identification and availability of relevant data, and any legal documentation. These steps are important to ground a project’s design and research question in the available array of human and material resources and to form expectations about the potential research outcomes that are informed by awareness of the quality and availability of data. As previously mentioned, analysts work very closely with clinical researchers to define research questions that closely align with the available methods and data. For instance, in the case of research on diabetes or stroke, the analyst may ask: are we analysing GP data, hospital data, or prescriptions? Then, the analyst also helps the researcher to think about what clinical informatics codes should be parsed to select instances of the phenomenon of interest, and prepare a ‘coding list’.

Researchers can find this process difficult because they are not allowed to conduct a preliminary assessment of the data, but also because they are sometimes less experienced in working with specific routine data than are the analysts. In this sense, the analysts’ effort to define the research topic proves helpful to setting up a feasible investigation. At the same time, clinicians bring in-depth experience of the context in which much routine data were generated, which helps to avoid unwarranted assumptions in data analysis, and to externally validate the results. For instance, a clinician tends to be better aware of how eventual financial incentives for GPs to record certain conditions can offset reporting of the phenomenon, or that cautious diagnostic behaviour associated with conditions such as depression causes it to be under-reported as diagnosis. The dialogue between SAIL analysts and prospective researchers can take several iterations until an agreement is reached that aligns all perspectives on a research question, a set of rules for sample selection, and the methods of analysis.

Researchers then submit a data access application which, following the collaborative interaction with analysts, is much more likely to be accepted by the governance panel. The application undergoes two tiers of information governance procedures: approval by top management and review by the IGRP. The first is aimed at committing human and technical resources according to schedule. Then, at the IGRP stage, panel members evaluate the motivations, methodologies and aims of the project, as well as the risks and feasibility of their proposed study, and the subset of SAIL data to which researchers request access. Applicants, IGRP members and SAIL analysts engage in negotiations around what research may be feasible given the available data sources and what constitutes sensible, reliable and safe research given the nature of the data and of the research questions at hand. Submissions need to specify precisely what variables are required, and what researchers will do with them. The panel pays special attention to the trade-off between disclosure risk and the epistemic gains of working with the requested variables: If some variables are very specific, they might disclose the identity of the patient.^11^ In some cases, researchers can be asked to find workarounds at different levels of aggregation. Despite the potential for delay, the review can also help the researchers to formulate a better plan for a time- and resource-efficient research project, as the parties involved in the review offer evaluation and constructive criticism about the intentions and potential gains of the research, and expert knowledge of the state of the available resources and the risks that can be foreshadowed in attempting the proposed data repurposing. In this respect, the process depends on the datasets involved.

The delegation of data governance to the IGRP varies depending on their licensing, with accordingly different procedures. SAIL hosts data of three different kinds: core datasets (e.g. Primary Care GP, Patient Episodes Database), restricted datasets (e.g. Cancer Registry for Wales; Congenital Anomalies database), and project-specific datasets. The governance of core datasets is entirely delegated to the IGRP. Restricted datasets are datasets for which the individual data provider wants to vet any data access application. This review is in addition to and independent from SAIL’s own IGRP review, and can require the project to be delayed and reconsidered when concerns are raised. Project-specific datasets are datasets provided by researchers who own them (e.g. clinical researchers who have collected their own data through standard protocols and ethics clearances) and who are interested in linking them to SAIL datasets in order to pursue their specific research question. By offering different tiers of participation in the data sharing infrastructure, SAIL accommodates varying levels of concerns and scrutiny, to make it possible for otherwise inaccessible research resources to be mobilized.

### Controlled research environment

**Figure 1.**
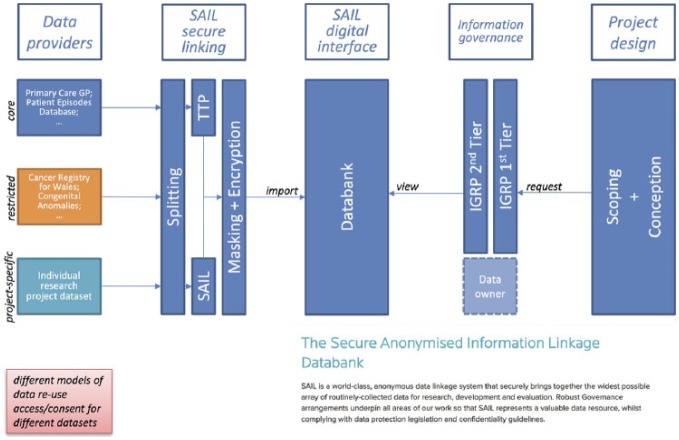
A summary of the sequence of processes that take place to make the data travel from data provider to end researcher in SAIL.

Third, the aim of catering to the needs of a global research community (not only Swansea’s), while controlling for the security of the research from both an epistemic and an infrastructural perspective, led SAIL managers to develop a secure analytical environment for researchers to access and analyse data. SAIL developers constructed a virtual environment within which the data are provided together with the software needed for analysis, such as statistical analysis packages, word processing, spreadsheet and programming languages. Systems administrators activate the virtual environment while the analyst prepares the data extract (see Figure 1) by selecting the data, masking unique data points^12^ and encrypting the demographic variables a third time.

The technology making up this environment has been designed to constrain human agency, ensure compliant behaviour, and create an audit trail. Some software functionalities (e.g. copy and paste) are removed to disallow users from exporting data. Similarly, the ability to copy files is disabled. Only derived aggregate information (e.g. tables with results of statistical analysis) can be exported at the end of the research project, for inclusion in research outputs, conditional to review and approval by the assigned ‘data guardian’ – a senior analyst. Researchers thus access the data extracts to which they have been granted access to only remotely and virtually. Through this virtual environment, data access is strictly regulated via automated restriction to specific users, times and resources; user behaviour is monitored and can be audited at any time; and functionality that constitutes an unacceptable IS risk is restricted. In this way, SAIL can guarantee to data providers that their ‘data never leave the system’, an important guarantee given the paramount importance of trust in data sharing.

SAIL is a full-fledged research infrastructure. It has evolved over time through negotiations that connect the requests advanced by researchers seeking to create new knowledge, and the concerns, conventions and regulations that require research projects accessing individual level data about the public to be trustworthy and accountable, following best practices and applying high methodological standards. The complex solutions that SAIL has developed over time are the result of repeated interfacing, and exemplify how the construction of strong infrastructural security measures occasions the exploration and development of solutions to improve and maintain the epistemic value of the data. The deep knowledge of datasets’ potentials and limits that analysts make available to researchers would be useful even in the scenario of a re-use infrastructure that did not need to fulfil patient protection requirement. Epistemic and infrastructural concerns continuously shape choices about the resources, the time and the data that are provided to researchers in the context of each project. SAIL’s chief technology officer stressed how, in designing the virtual environment, a top priority was ‘not to disadvantage researchers’. Both EIS and IIS are paramount for the sustainability of SAIL, and determine the specific solutions adopted to safeguard data access and use.

Moreover, the convergence of EIS and IIS is illustrated by the design of the SAIL pipeline and information governance processes. Incoming datasets are checked to satisfy quality standards; data are prepared to maximize their usability as evidence, through the crucial role of the analysts and their curation and analytical work aimed at mitigating risk and optimizing data re-use; bespoke resources are developed to control user agency while providing cutting-edge analytical tools and technologies; and each project is assessed, through the scoping and governance reviews, with respect to its scientific potential, the risk and benefits involved, and its feasibility in light of available knowledge about the quality of the data and the fit of the methods selected for their analysis. In particular, EIS concerns with the soundness of research questions, the adequacy of data sources and the soundness of methods of analysis constitute essential components of the risk/benefit analysis at the core of research governance. The interplay of IIS and EIS concerns thus provides a valuable point of reference for developing methods and roles that advance best practices in the re-use of complex, diverse, very large and often messy routine datasets.

IS measures arguably benefit the long-term reliability of the knowledge being produced through SAIL. Nevertheless, they also fuel uncertainties which can shape the trajectory of a project, occasioning delays, changes in the execution or shifts in ways of working with data, software or colleagues. These effects are most visible in the short term and from within the frame of the individual research project; they are likely to cause frustration among biomedical researchers under pressure to produce results to match pre-defined schedules and funding agreements. However, the epistemic costs of implementing secure solutions can be balanced by gains of the same kind, generated by deeper collaboration, enhanced trust in data collection and analytic tools, and relations of interdependence and scrutiny among data stewards and researchers.

## The Medical and Environmental Data Mashup Infrastructure

Our second case consists in a health data linkage infrastructure that has just gone through its first phase of development through an initial, three-year funding stream (2013–2016). MEDMI was funded by the Medical Research Council and the Natural and Environmental Research Council with a budget of approximately £1 million, and brings together researchers from the University of Exeter Medical School, the London School of Hygiene and Tropical Medicine, the Met Office and Public Health England (PHE). MEDMI’s signature characteristic is its remarkable interdisciplinarity. The project brought together partner institutions with very different kinds of expertise and resources in order to facilitate research at the crossroads between climate and environmental sciences, epidemiology, genomics and public health. MEDMI aimed to: 1) source datasets from these different epistemic communities, and develop an infrastructure to make them available for re-use by interested researchers, 2) develop methods and tools to link and analyse the relationships between complex meteorological, environmental and epidemiological data, and 3) foster research that can demonstrate the usefulness of MEDMI methods and tools, which was done by sponsoring three demonstration projects focused on different areas of research on the links between the environment and human health (Fleming et al., 2014, 2017) Like SAIL, MEDMI is a data linkage infrastructure, whereby different datasets are linked or made linkable to make it possible for researchers to combine and compare patterns, to produce new evidence for claims about imputed relations between environment and health.

The project had an uneasy start, due to a combination of optimistic expectations about procurement of technological, human and data resources, and complexities in the coordination between partners that were compounded by a lack of specification of milestones needed to reach end goals. Coordination was made harder by the fact that PHE was undergoing significant organizational restructuring, as it had just been formed through a merger of the former Health Protection Agency, the original MEDMI Partner, and other governmental agencies in the Department of Health, with consequent and enduring confusion on duties and responsibilities. Furthermore, the relatively small size of the grant combined with the number of participating partners meant that roles and responsibilities were spread across partner institutions, with several senior investigators appointed mainly for oversight functions, and only one fulltime and two part-time fellow researchers appointed to conduct the core of the infrastructure development and data analysis work. Among the technical issues that delayed early progress was the problem of figuring out the location for the servers; for security reasons, cloud architectures had to be discarded in favour of a server hosting on-site on the Exeter campus.

### Battling with IIS

An aspect of infrastructure development that became the source of several complications was relative to the lack of a strong ‘data custody proposition’ through which data providers would give custody of data to MEDMI. This turned out to be necessary for MEDMI to be seen as a trustworthy custodian, able to govern the re-use of datasets in a way that protected patients and the standards of research at the same time. Early attempts to source the indispensable datasets of human health data hit dramatic delays, as the involved parties struggled to communicate clearly with each other over the concerns and consequences of different possible scenarios of data licensing^13^ and republishing.

Early on, the partners expected that the project would deliver a linkage system, provided with data, that could be used to execute three demonstration projects. The envisioned infrastructure would also include a public website to make available some linked datasets for integrated, in-browser, analysis. To achieve this, the weather, climate, environmental and health datasets to be linked had to be made available to MEDMI developers and uploaded to the server. The provision of core datasets was the responsibility of the Met Office, which would supply weather, climate and other environmental data, and of PHE, which would supply health data. However, the first batch of human health data was loaded on the server only at the end of the project, three years after the official start.

Negotiation for health data access at PHE developed slowly, due to the need to define re-publishing conditions under which MEDMI would be able to share data with third-party researchers. To make the issues more complex, while some of the datasets were directly owned internally, others had to be negotiated with relevant third parties such as the Office for National Statistics or The Phoenix Partnership (TPP), a software vendor. The MEDMI partners and the data owners knew of no existing methods and legal agreements that could immediately fit. The lack of early and strong specification of end features and workflows of the infrastructure, including a systematic approach to protecting confidentiality and guarantee the security of data, contributed to the protraction of discussions. Negotiations centred on patient protection bore deep implications for research processes and their features, including the choice of methods and operations to prepare and link the data for re-use, and the scientists involved in executing these operations.

For example, due to the lack of a data custody proposition, the UK Biobank refused to share their source data (to be linked in MEDMI with other MEDMI data). They instead offered to link the data themselves, which would involve receiving environmental data from MEDMI and sending derived datasets back, and to providing access to the linked data only to specific, named MEDMI researchers. UK Biobank staff saw this as a way to retain control of the personal data entrusted to them by individual participants and medical institutions, and thus guarantee that there would be no breach of security – both in the sense of preventing data loss (IIS) and in the sense of avoiding misinterpretation of the evidential value of the data (EIS). This proposal could not be accepted by MEDMI: It would undermine the epistemic goal of MEDMI to enable interdisciplinary work on environmental and health data, and would thwart its development as a reference data linkage platform. MEDMI staff adapted aims and expectations around the data resources that were readily available.

Largely as a consequence of the lack of a clear data custody strategy and related delays, MEDMI drifted away from its initial concept and was split into two separate components: open and restricted access resources. Given the security risks involved in handling human health data, and the related difficulties in data licensing negotiations, it became clear that providing a publicly accessible data interface, comprising a set of tools for statistical analysis running in a browser window, was not a realistic prospect. As demonstrated by the case of SAIL, the technical and long-term resource commitments needed to achieve this functionality in ways that were both infrastructurally and epistemically secure were overwhelming. The scope of this MEDMI technology was consequently limited to demonstrating what new interfaces for environment–health research infrastructures could be capable of, once trustworthy security was granted. One strand of the project thus focused on making the technology run on a test dataset that would be cleared for open republishing.

A second strand of the project concerned the components of the infrastructure that involved sensitive human health data, and focused on facilitating data access, linkage and analysis under conditions of restricted access. This part of MEDMI included some of the browser-based analytical tools and a server-based library of Python^14^ modules for flexible data linkage, which users would execute via command-line programming. To make this work, a new set of information governance solutions and policies was drafted that made it possible for individual researchers to apply for data access and discuss these requests with the original data providers, thus maintaining a tight control over who was given access to health data, and for which purposes.

Two years into the project, MEDMI infrastructure had thus been substantially re-thought to respond to emerging concerns over potential unauthorized access and data misuse. To become a trustworthy data custodian, MEDMI had to find ways of securing data storage, access and re-use from both an epistemic and infrastructural viewpoint. The initial, open concept immediately suffered from the pressure of feedback from actors external to MEDMI, because of the thinness of its IS strategy, and progress was delayed by related issues, ranging from cloud architecture to human health data licensing. The epistemic goals of specific features and technologies changed as result of unaccounted factors, including access control requirements and overwhelming infrastructural security complexity; while the implementation of the restricted access part of the infrastructure that offers researchers powerful linkage tools (to conduct data linkage with at high scientific standards) required the development of information governance procedures to move forward.

### Implications for knowledge production

These delays led some of the demonstration projects research to be performed on data outside of the MEDMI infrastructure, and others to work with proxy measures that were imposed by patient protection requirements. These modifications affected knowledge-making processes and outcomes in ways that could, prima facie, be seen as favouring IIS to the expense of EIS. For example, one of the demonstration projects focused on investigating the seasonality of pathogens responsible for a range of human health infections, including families of food poisoning agents *salmonella* and *campylobacter*. To this aim, the project planned to investigate potential correlations between the locations of specific cases of infection and variations in weather and environment at those locations (e.g. if weather is warmer than average, some bacteria can thrive). IIS concerns hampered this plan, as using precise data on patient location (such as for instance the postcode of their home address) would increase the risk of patient re-identification, particularly in scarcely populated areas. As proxies for patient locations, the researchers thus settled for the postcodes of the laboratories where patient specimens were examined. This arrangement was viewed as epistemically problematic, since patients may sometimes live relatively far away from labs, thus compromising the reliability of such location data for the purpose at hand. Some researchers feared that security measures had compromised the value and reliability of the data as evidence for disease seasonality – thus, in our terms, highlighting both the distinct characteristics of IIS and EIS, and the existence of a conflict between the two.

IIS concerns did shape the resolution and definition of geographical space upon which analyses could be conducted, thus affecting the type of research questions and methods that could be applied to the data in question.^15^ Nevertheless, EIS was not lost or compromised as a result. Rather, researchers validated the new method of tracking location as yielding comparable results, thus mitigating fears that the resulting inaccuracies would compromise the reliability of the analysis. Faced with the constraint, researchers were able to conduct the project in a way that held EIS and IIS under control. The benefit was not limited to the individual project, as the solution encouraged further research on alternative locality measures and their underpinning assumptions and methods of validation. This can foster the effective use of different types of location data in the future, and improve researchers’ awareness of the conceptual and technical pitfalls of using postcodes as proxies. This example fittingly demonstrates how EIS and IIS concerns need to be balanced in the course of any investigation. The process of accommodating both sets of demands affects the direction and outcomes of biomedical research in ways that researchers may not have predicted – and yet, the overall effects of these modifications are not necessarily damaging to research outcomes, and in fact can stimulate broader scrutiny of the long-term risks of and opportunities for reusing certain types of data.

Experiences such as this allowed MEDMI staff to develop a better awareness of the wide range of different needs, standards, working hypotheses and designs, and kinds of data that researchers work with. The result was a total re-conceptualization of the data linkage methods to be adopted to enable the three overarching goals of MEDMI. In its planning stage, MEDMI was devised as utilizing a ‘universal’ approach centred on a single, standardized method for combining datasets with one another. This was viewed as increasing the usability of the infrastructure, however it implied black-boxing many of the assumptions underpinning data linkage to favour smoother and easier consumption by researchers with limited IT skills. Once confronted with IS concerns, MEDMI shifted instead to a ‘situated’ approach relying on a flexible library of computational modules. This would require more commitment and learning by the user, who would need to become aware of – and explicitly decide about – the parameters, assumptions and trade-offs through which datasets of very different origins, formats and resolutions were to be combined.

While more complex to develop and use, the new, more flexible system has a superior approach to information security. On the one hand, it enforces IIS by making the system able to work with data shared with different levels of access and protection requirements. On the other hand, it enhances EIS by allowing to tailoring searches and linkage parameters to the situated needs of researchers working on a specific project, thus facilitating critical thinking on how data should best be interpreted in relation to specific research questions and situations. For example, data linked through MEDMI have varying levels of spatial resolution (consequence of the different methods and situations of data generation and original use, as well as the different security regimes imposed on sharing), and MEDMI staff decided that decisions about how to combine and interpret such differences were best left to the researchers in charge of each specific projects.

Certainly, the shift from universal to situated data linkage was costly, both in terms of resourcing by MEDMI staff and in terms of the overall user-friendliness of the system. It compounded the disconnect between the public-facing demonstrative tools running on test data and the research-grade infrastructure; and delivered a technology with a much steeper learning curve for the end user. However, the new linkage method was technically superior and more powerful; it was able to flexibly support different pieces of research building from different sets of definitions of spatial aggregation, and diverse working assumptions and hypotheses; and it made the infrastructure adaptable to the ever-changing requirements of infrastructural security, and specifically the evolving standards of patient protection.^16^

On the basis of the new approach, in 2015 a programme of mini-grant funding was opened to researchers from the participating institutions, aiming to encourage the exploration of the MEDMI data and linkage tools through small pilot projects that could be executed in a few months. This was a resilient and emergent response to the challenges that had punctuated the development of MEDMI. A diverse set of projects were funded, some successfully developing into publication. Despite the severe challenges confronted throughout the project, MEDMI still reached the end of its funding stream producing more research outputs that had been initially anticipated.

## When conflict arises: IIS and EIS in a data journey across MEDMI and SAIL

With the preceding material, we hope to have demonstrated how EIS and IIS concerns can be aligned to the advantage of both researchers and data infrastructure managers, while at the same time fostering the long-term sustainability, ethical standing and reliability of data and the knowledge for which they are used as evidence. To avoid being misinterpreted, we also want to stress, however, that these two forms of IS are not always treated consistently, and many factors can contribute to the widespread perception of IIS as being overly restrictive and damaging the epistemic value of data for research. A useful example of this comes from one of the small pilot projects funded in 2015–2016 by MEDMI. This is also an interesting instance insofar as it involves a collaboration between MEDMI and SAIL, thus providing a link between our two cases.^17^

The pilot project in question explored the influence of exposure to green spaces on childhood obesity, with the aim to validate previous findings that suggested positive correlations between health outcomes and green/blue space proximity. It involved the extraction of human health and social data from SAIL, including height, weight and body mass index collected in primary schools by the Wales Childhood Measurement Programme and the Welsh index of social deprivation, and their linkage with environmental and weather data in MEDMI. Given the sensitivity of the data taken from children, a condition for the extraction was data suppression, through ‘masking’, of low individual counts to protect unique data combinations and avoid re-identification. To this aim, data masking involves raising the number of a low count category to 5 (or higher, to add additional protection if higher risks are estimated) and adjusting total counts accordingly, so that a person accessing the category data cannot easily connect the information to a specific individual. Another condition for the extraction was the use of spatial aggregation of units of analysis at the resolution of Lower Super-Output Areas (LSOA), a relatively coarse (yet standard) method to split a geographical area by population size, which means that environmental data would also have to be adjusted to allow the linkage.^18^ Variables were then derived to estimate, for instance, the amount of greenspace linked to a LSOA and the proximity to coastline. The spatial segmentation and resolution of LSOAs were primarily chosen to align with the variables used by the social deprivation index.

Unexpectedly, adopting LSOA resolution and applying the IS regime resulted in drastic information loss. Since LSOA populations are relatively small, and the subdivision of children further divided in obesity-related bands further selects increasingly smaller subsets, a high proportion of the data were suppressed. Children falling in high BMI bands (equated with obesity for the purpose of the study) can be relatively rare in some LSOAs, and their low counts hit data-masking thresholds. Most importantly, the suppression was not randomly distributed, as it hit mostly in the scarcer tail end population categories (such as obese children, as opposed to non-obese; and rural, as opposed to urban), thus confounding the derived relationships between obesity and green space that were the centre focus of the analysis. The resulting dataset, with up to 50% of non-randomly suppressed data, was highly biased and made it impossible to disentangle ‘what makes certain areas healthier than others’. It should be emphasized that the problem was not due to the resolution of data at LSOA, since the choice of aggregating units of analysis at a coarser spatial resolution produces higher denominators (‘counts’), and this can reduce the amount of suppression (as well as of random fluctuations) in the observed statistical relationships. Instead and crucially, since data masking is applied as soon as the human health data are taken out of SAIL, the heart of the problem lay in applying data masking *before* analysis (to allow data to be analysed in MEDMI) – as opposed to *after* analysis (if the analysis were conducted within SAIL itself). Within SAIL, it would have been possible (once approved by information governance review) to conduct research at individual rather than aggregate levels, thus side-stepping the problems generated by masking.

Indeed, in their report back to MEDMI, the researchers who undertook the pilot project observed how the suppression made necessary by security requirements made the analysis insensitive to an association between rainfall and obesity that is otherwise likely to be detected. Further, they noted how it would not be possible to store the data exported from SAIL in aggregate form within MEDMI, due to licensing and confidentiality restrictions. As an alternative path for future inquiries, the researchers suggested that rather than extracting human health data from the existing anonymized infrastructure SAIL, to be linked with environmental data in MEDMI, environmental data should be imported for linkage into the secure virtual environment of SAIL, within which analyses can be conducted at individual household resolution. If linkage is conducted at the site where the most confidential data are held (in this case, SAIL), the information loss would likely be smaller.

This example shows the two forms of IS working at cross-purposes, with requirements for data suppression responding chiefly to IIS, making the analysis insensitive and unreliable, and thus insecure from an epistemic perspective. The pilot project in question was severely compromised and ultimately failed as a result of the implementation of IIS measures. This is the kind of example that makes biomedical researchers fear IIS measures as obstacles to explorative research and effective discovery, and cast IIS measures as conflicting with EIS concerns around making the best use of available data. At the same time, the researchers’ suggested solution to the issue at hand points us, once again, to the possibility – and epistemic advantages – of re-aligning IIS and EIS concerns. It also points us to the significance of the availability and choice of research environment and infrastructures towards making research successfully secure in both respects. Reconciling IIS and EIS to conduct this kind of research is by no means impossible, but it does require a re-arrangement in the institutional responsibilities, expertise and funding arrangement of the research team that is strategically directed towards facilitating data analyses that are both epistemically meaningful and safe for human subjects. This re-arrangement could not realistically be supported within the time-line of the pilot, nor possibly even within the scope of a project such as MEDMI. What this example shows, in sum, is how the design of the pilot did not take into account the implications of linking SAIL and MEDMI data, nor did it recognize the ways in which such linkage could be organized in order to prevent concerns around data confidentiality and misuse (namely, by shifting the site of linkage from MEDMI to SAIL). Rather than IIS being a problem in and of itself, therefore, the emergence of a conflict between IIS and EIS in this case highlights how sustainable, socially responsible and epistemically trustworthy knowledge production requires not only the development and implementation of apposite, sophisticated systems of data governance and interpretation, but also careful planning of any specific instance of data re-use – so that these systems are properly exploited.

## The role of information security in biomedical data journeys

The power of data to support a range of evidential claims is highly dependent on the specific situation of use and the aims and skills of those who handle them (Borgman, 2015; Kitchin, 2013; Leonelli, 2016a). Hence, a condition for the successful and repeated re-use of data is that, during their movements and transfers from system to system and between situations of use, data remain credible and usable, such that they retain the capability to support epistemic claims throughout the journey. We view the interplay of infrastructural and epistemic forms of IS as the core issue underpinning the credibility and trustworthiness of research processes centred on the re-use of biomedical data. Both in SAIL and in MEDMI this interplay shapes scope and aims of research projects, the selection of relevant datasets, the choice of methods of data integration and analysis, the order and dependencies within the research workflow, and the evaluation of a research project’s risks and benefits. Through our empirical narratives, we have shown how IS regimes affect and enable the scientific process beyond mainstream critiques that see it as cumbersome, alienating and constraining. We have also seen how many factors – some of which are not under the control of researchers – need to be taken into account, which may slow down and sometimes upend the start and the progress of research. However, this is not necessarily an insurmountable obstacle to research on datasets.

Instead, IS is critical to the enactment of scientific processes as socially embedded, distributed, cooperative, situated action. IS is critical to the development of methodological, organizational and technological solutions that are not limited to merely satisfying patient protection concerns in the short term, but also introduce innovations in the structure of complex collaborative action that facilitate trustworthy data linkage and re-use research in the longer term. Agreement on IS regimes among the parties involved in overseeing data re-use and fostering a culture of trustworthy practices includes the sharing of relevant in-depth expertise, methods, software and data resources, which make data re-use science not only possible but also resilient and responsive to social concerns around data confidentiality and potential data misuse. Through the design and implementation of governance procedures, organizing protocols and information technology, IS regimes provide methods to streamline and simplify the complexities of devising ways of working with data securely and safe from accidents and other unwelcome consequences of data circulation gone wrong. Information governance processes designed to review and authorize every stage of a project (from its inception to the eventual publication of results) help researchers to critically question their assumptions and expectations with regards to data sources, formats, necessary metadata and potential uses, and the wider context of their research. The support of expert analysts allows bootstrapping the learning process that is inevitably involved when attempting to re-use a preexisting data resource with a complex background history.

Our two cases show how innovative research methods, valuable within and beyond the research community, are created and developed through a productive alignment of responses to concerns of both EIS and IIS. The tensions usually associated with IS measures are dependent on a broad set of concerns, which are linked to the underdetermined relation between data infrastructures (and related expertise), the diverse settings in which datasets are generated, the wider landscape of governmental policies, institutional and industrial ecologies, research trends, funding streams and scientific communities (especially in interdisciplinary projects).

These conditions affect innumerable situations of data re-use. Our analysis illustrates how IS regimes enable data re-use not only from an infrastructural and organizational perspective, but also from an epistemic perspective, for instance by sharing well-tested methods, resources, and specialized expertise and knowledge about limitations and opportunities that a dataset may present. We argue that EIS and IIS are mutually constitutive. The implementation of bespoke solutions with the aim of addressing infrastructural security concerns contributes to scrutinizing, improving and shaping research questions, design and methods. It provides deep expertise about qualities and limitations of the data, and resources for data analysis. It makes available tested, state-of-the-art methods and resources that relieve researchers from the pressure of designing secure and accountable protocols of their own, and rationalizes the deployment of scarce resources to optimize re-use of the data. On the other hand, the development of strategies and resources to improve the epistemic security of the long and multi-layered processes of data sourcing and re-purposing contributes to the maximization of research opportunities, and positively influences the valuations of risks and benefits that are critical for a sustainable and long-term operation of secure infrastructures.

Since they are mutually constitutive, in which way is it helpful to distinguish between epistemic and infrastructural forms of IS in the first place? We argue that the distinction matters, particularly in those situations where EIS and IIS concerns diverge and find themselves working at cross-purposes. We have documented frictions and uncertainties borne out of unsuccessful attempts to align the diverging demands of different sets of security concerns, and the failure to take advantage of existing methods and approaches from the start of the research process. In this respect, we observe that an IS regime shapes the scientific process both purposefully and contingently. It does so purposefully, by encouraging researchers to challenge preformed expectations, anticipate public reception, test methodological robustness, and by providing analytical tools, expertise and resources that help can ensure research processes are secure and meet stipulated standards. And it does so contingently, through ongoing adaptation to the ever-changing characteristics of research conditions and outcomes, as well as relevant governmental policies, regulatory regimes, insurance requirements, funding sources and technological developments.

Flexibility and the ability to adapt are particularly important given the shifting and sometimes unclear interdependencies involved in resourcing, managing and planning for the required infrastructures. In the case of MEDMI, negotiations of legal and organizational arrangements absorbed great amounts of time with significant implications for the long-term evolution of the project. At the same time, vulnerabilities and threats of critical severity were continuously exposed, and at times hijacked the technical development of the infrastructure. Values and views on data re-use research are sensitive to changes in scientific standards and methodological developments, and the never-ending evolution of the ‘threat landscape’. Worries around the costs of IS tend to canalize and highlight this broader set of concerns.^19^ As Burton et al. (2015) pointed out, infrastructure projects are continuously tested for these potential tensions, and for this reason need to adopt an anticipatory, dynamic approach.

The minimization of frictions and uncertainties is of paramount importance for the long-term operation and sustainability of a secure infrastructure (Tempini, 2016). Both EIS and IIS are needed for research infrastructures to become trustworthy custodians of data. SAIL management often described the core of their activities to us as acquiring access and enabling the re-use of datasets that are otherwise left dormant and unused. They stressed that the survival of their entire infrastructure does not depend on the untenable guarantee of infallible security, but rather on successfully demonstrating trustworthiness to their many relevant audiences. The development of sophisticated secure virtual environments is thus extremely valuable to securing data provision in SAIL. Similarly, in MEDMI, health datasets could not be successfully sourced without compartmentalizing the infrastructure to offer different resources and capabilities depending on the degrees of access control involved, and drafting information governance processes to demonstrate that appropriate surveillance and oversight capabilities were in place.

Ensuring that the organizational, social, ethical and legal conditions of IS are satisfied constitutes an unescapable pre-condition for an epistemically secure science, rather than a limitation. IS must be built into the system despite the counter-balancing constraints that it may impose. Even in cases where IS regimes may block or severely challenge one or more individual research projects, the question is whether they increase the overall sustainability of a given research infrastructure and related systems for data re-use, especially its ability to survive changing levels of public scrutiny. Our analysis shows that IS regimes contribute resilience to epistemic processes involved in re-using biomedical data, even in the face of occasional attempts at data re-use that fail.^20^

Such mobilization and repeated utilization of datasets goes a long way to achieve what the open science movement is advocating, but the means are different – and much more sophisticated – from simply making data widely accessible. As we have seen, IS can be both a constraint and an enabler to research. The appropriate implementation of both EIS and IIS within data re-use infrastructures is crucial to facilitating the production of new biomedical knowledge in ways that preserve its integrity, quality and social accountability. It is also a necessary starting point for experimenting with innovative data governance concepts that may overcome the tension between individualized consent and public good (e.g. Prainsack and Buyx, 2017).

## Notes

1. See for instance Harris et al. (2016) and the recent double special issue on ‘digital circulation’ in Tecnoscienza (Balbi et al., 2016, 2017). In the UK, the NHS regularly tops data breaches rankings (Gold, 2010).

2. The National Information Assurance Glossary edited by the US Committee on National Security Systems (2010) defines IS as ‘the protection of information and information systems from unauthorized access, use, disclosure, disruption, modification, or destruction in order to provide confidentiality, integrity, and availability’ (p. 37). As such it continues a long-standing trend defining IS as involving ‘CIA’, or ‘Confidentiality, Integrity and Accessibility’. The 2016 version of the reference standard ISO 27000 endorses a similar definition of IS as the ‘preservation of confidentiality, integrity and availability of information’ (ISO/IEC, 2016: 6), and reference handbooks for practice follow suit (Calder and Watkins, 2008: 6). This definition has also been accepted by philosophers and social scientists discussing matters of information or computer security (e.g. Nissenbaum, 2005). According to this framework, to achieve IS involves control over three interdependent dimensions: the *accessibility* to the data in question, the level of *confidentiality* and related protection with which sensitive information is invested, and the *integrity* of the data – that is, the extent to which the data being disseminated are preserved from degradation, loss or unintended transformation.

3. This holds even when accidents originate from within the organization (for instance, an officer losing top secret information on public transport): The event acquires significance because of the wider context (i.e. the possibility that someone can get hold of the information, and make use of it). The informatics literature on security, we argue, needs reflexive investigation of the role of information security practice itself in data processing for research purposes (Tempini, 2016).

4. As well-documented by science and technology studies scholars and within current debates around ‘open science’, the strategies used to collect, manage and disseminate data play a crucial role in shaping data interpretation and re-use (Boulton et al., 2012; Bowker, 2000; Demir and Murtagh, 2013; Edwards et al., 2013; Hilgartner, 2013; Hine, 2006; Kitchin, 2013; Leonelli, 2016a; Stevens, 2013; Tempini, 2017), and the political economy in which they are embedded (Mirowski, 2018; Tempini and Del Savio, 2018).

5. This research is part of a broader study encompassing detailed investigation of several different data journeys. More details about this work are available on the Data Studies website (https://datastudies.eu/) and the edited volume *Varieties of Data Journeys* (Leonelli and Tempini, forthcoming).

6. Transcripts that participants have agreed to publicly disclose are openly accessible within the Exeter Data Studies data collection hosted by Zenodo (URL: https://zenodo.org/communities/datastudies/).

7. Health and Care Research Wales is a department of the Health Directorate of the Welsh Government, and is tasked with supporting health and social care research in Wales using funding from the national (devolved) healthcare budget.

8. De-identified demographics include: an encrypted number to SAIL that acts as identifier (called the Anonymised Linking Field, ALF), and demographic data transformed to lower resolution (date of birth becomes week of birth, postcode becomes Lower Super Output Areas – a much coarser administrative geographical unit, etc.).

9. Since, for confidentiality requirements, no actor holds the entirety of the data, it is very difficult for SAIL and its partners to share definitions of the problem situation, triage potential causes, and coordinate corrective action. At one point, SAIL stopped receiving data from one third of the connected GP practices. Reconstructing what part of the data anonymization process had broken down took more than a year.

10. As more records are linked with one another and re-used, the risk of re-identification (also referred to as ‘deductive disclosure’) creeps up: if enough data are available at once, anonymized demographic can be re-identified. For this reason, data are encrypted a third time, each and every time they are made available for a specific research project, to protect from misuse of the data by researchers who access SAIL data through multiple projects (or by others gaining access researchers’ systems or credentials in turn).

11. Panel members will check, for instance, if the research is focusing on a unique condition, on individuals of a narrow age group or from a specific geographical location.

12. Masking is a technical term for the operation through which low count data points, whose sharing is considered a re-identification risk, are swapped for a higher default value. For instance: if one shared aggregate data about social groups from a certain rural area, including about a group of one (n=1) of obese children, the group data could be easily attributed to the child, thus disclosing information. The group data can be masked by sharing an artificial, higher group count (n=5), thus making inference to individuals less meaningful.

13. Licensing issues are often featured in discussions about potential sources of operational risk, but often are not understood as a security risk. However, as we will show briefly, in our framework of IS, licensing negotiations feature as a point of convergence of security concerns which can have far-reaching consequences for epistemic and infrastructural IS. For this reason, licensing features in our narrative precisely because of its role and implications related to security.

14. A high-level programming language that is a very popular choice of scientists and developers for data analysis and mining work.

15. For a detailed analysis of different conceptualizations of locality in this case, and their impact on data linkage, see Leonelli and Tempini (2018).

16. A particularly useful element given the ongoing implementation of the General Data Protection Regulation in the UK and Europe, which has transformative implications for health-related research (Leonelli, 2017).

17. Incidentally, we were not aware of this when we first selected them as central focus for our research, and at the start of fieldwork, the project was not yet completed.

18. Approximately 1500 residents define each specific LSOA, with some variation; this results in greatly varying surface areas, as LSOAs in densely populated urban environments are much smaller than LSOAs in the most rural settings.

19. Underscoring this point, the PI of a large computational data center that we interviewed in 2016 emphasized the uncertainty generated by non-technical factors of IS:
the technical implementation has probably been 10% of the work, and the legal wrangling and security documents, and the number of time it’s been around the various IS officers’ desks - and not just at our institution, but of our potential partner - has just been absolutely astounding. It’s easily 90% of the time and work, and we’re still not done. (PI_16_A)

20. A parallel argument can be made for the role of ethics in data infrastructure, as discussed by Leonelli (2016b).
